# The golden hour: the feasibility of a quality improvement project to decrease the time to antibiotic administration in children with cancer and febrile neutropenia in Jordan

**DOI:** 10.3389/fped.2026.1920472

**Published:** 2026-10-28

**Authors:** Hadeel Halalsheh, Ghada Abu Alrub, Islam Kallab, Nimeh Al-shawabkeh, Ahmad Suleiman, Tamara Dabbagh, Tasneem Dajani, Iyad Sultan, Faiha Bazzeh

**Affiliations:** 1 Department of Pediatrics, King Hussein Cancer Center, Amman, Jordan; 2 Department of Pediatrics, The University of Jordan, Amman, Jordan; 3 Quality Office, King Hussein Cancer Center, Amman, Jordan; 4 Department of Nursing, King Hussein Cancer Center, Amman, Jordan

**Keywords:** febrile neutropenia, golden hour, low- and middle-income countries, Middle East, quality

## Abstract

**Background:**

Febrile neutropenia (FN) is a potentially life-threatening condition in pediatric oncology; early antibiotic initiation is associated with improved survival. A quality improvement (QI) project was implemented in the Pediatric Emergency Room (ER) to improve the timeliness of antibiotic administration.

**Methods:**

Starting in July 2019, the project was designed using the Find, Organize, Clarify, Understand, and Select–Plan, Do, Check, and Act (FOCUS–PDCA) framework. We aimed to give antibiotics within 60 min of ER arrival for children with suspected FN. High-risk patients received antibiotics without waiting for blood count confirmation. A structured staff education campaign was conducted over 6 months, and performance metrics were continuously monitored. Outcomes were compared with retrospective data from December 2018 to June 2019.

**Results:**

The pre-intervention cohort included 119 children with cancer, accounting for 158 FN episodes. From July 2019 to December 2024, during the intervention period, 697 children with cancer (median age 4.7 years; IQR 2.6–9.2 years; 54% male) presented with FN, accounting for 1,164 ER visits (median 1 visit/patient; range, 1–11). The median absolute neutrophil count (ANC) at presentation was 50 cells/*μ*L (IQR, 0–180 cells/μL). Median time from ER arrival to antibiotic administration decreased from 120 min (IQR, 97–143) at pre-intervention baseline to 50 min (IQR, 40–65) across the implementation period (*p* < 0.01). The time from antibiotic ordering to first dose remained stable (median 32 min; IQR, 25–40), while time from arrival to antibiotic ordering decreased from 53 to 15 min (*p* < 0.01). Compliance with the 60 min target improved from 22% in the pre-intervention period to a sustained 85%–90% range across 2021–2024, with the ≥90% target met in all 12 months of 2021 and compliance declining to 85.8% in 2024. The target was increased to 90% in September 2020.

**Conclusion:**

A structured Golden Hour QI initiative is feasible, effective, and associated with substantial and sustained improvement in antibiotic timeliness in the Middle East region, without additional funding, staffing, or technology. The core drivers of sustained improvement were automatic Category-A triage for children with cancer presenting with fever, pre-ANC antibiotic initiation for high-risk cases, and monthly audit-feedback cycles — all implementable within existing workflows.

## Background

Febrile neutropenia (FN) is the most common oncologic emergency, requiring prompt evaluation and treatment (1). Although most cases are low-risk, progression to septic shock, organ failure, or death can occur (2). Early initiation of empiric broad-spectrum antibiotics is therefore crucial. Timing matters: the “Golden Hour” concept holds that intervention within the first 60 min of presentation significantly reduces serious complications (3–5). Evidence supporting this threshold derives largely from adult studies; however, data in children with cancer remain limited (6).

Presentation-to-antibiotic (PTA) time — the interval from hospital arrival to first antibiotic dose — is the primary metric used to evaluate timeliness of FN management. The International Pediatric Fever and Neutropenia Guideline Panel recommends antibiotic administration within 60 min of presentation, a target informed by adult data showing that early ordering, even before laboratory confirmation of neutropenia, reduces serious complications (7–9). This threshold is frequently unmet in pediatric settings, prompting numerous quality improvement (QI) initiatives to reduce PTA time (10–14).

Reported QI interventions have clustered around four domains: expedited triage protocols, high-acuity flags to reduce door-to-physician time, standardized antibiotic order sets, and electronic medical record (EMR)-facilitated communication between triage nurses, physicians, and pharmacists (11, 15). Across these initiatives, median PTA time fell by 20–110 min depending on baseline performance, and the proportion of patients receiving antibiotics within 60 min improved from rates as low as 30%–55% at baseline to 80%–96% post-intervention (11, 15–17).

At King Hussein Cancer Center (KHCC) in Amman, Jordan — a 433-bed tertiary oncology center and the only institution in the country with a dedicated pediatric oncology emergency service — we launched the “Golden Hour” QI Project in July 2019, aiming to reduce PTA time to within 60 min of emergency room (ER) arrival for children with cancer presenting with FN. The center manages approximately 6,600 pediatric oncology ER visits annually, and the project required no new staff, technology, or budget: it relied on reclassifying children with cancer presenting with fever within the existing five-tier triage system, standardizing physician and pharmacist workflows already in place, and embedding monthly audit-feedback meetings into established departmental governance cycles.

## Patients and methods

### Study design and setting

This single-center before-and-after quality improvement study was conducted at KHCC, comparing a retrospective pre-intervention period (December 2018 to June 2019) with a prospective intervention period (July 2019 to December 2024), without a concurrent control group, and was approved by the KHCC Institutional Review Board. It was reported in accordance with the Standards for Quality Improvement Reporting Excellence (SQUIRE) 2.0 guidelines (18). The study dataset comprised ER visits of patients presenting with FN from July 2019 to December 2024.

### Definitions

Neutropenia was defined as absolute neutrophil count (ANC) < 0.5 × 10⁹/L. Fever was defined as a single oral temperature ≥38.3 °C or a sustained temperature ≥38.0 °C for one hour or longer. Golden Hour compliance required a PTA interval ≤60 min. Thirty-day mortality was defined as death from any cause within 30 days of the index ER visit. High-risk status was defined as any of the following: acute lymphoblastic leukemia during induction, intensification, or re-induction chemotherapy; recent fludarabine, cytarabine, and granulocyte colony-stimulating factor (FLAG) chemotherapy; Burkitt lymphoma; leukemia treated with corticosteroids; acute myeloid leukemia (AML) on active therapy; functional neutropenia (hematologic malignancies that impair phagocytosis even when the absolute neutrophil count is normal); the post-chemotherapy nadir period (7–12 days after the last chemotherapy dose); or a treating clinician's assessment that the patient appeared clinically unwell. High-risk patients received empiric antibiotics immediately, without waiting for ANC confirmation (Figure 1). The empiric regimen was guided by local microbiology and current pediatric FN recommendations (7). Amikacin was added for high-risk or clinically unstable patients, while glycopeptides were reserved for predefined indications for resistant or invasive gram-positive infection. These high-risk criteria were selected to identify patients at increased risk of profound or evolving neutropenia and rapid infectious deterioration and were consistent with pediatric FN risk-assessment principles (7). Monthly audit-and-feedback meetings reviewed key performance indicator (KPI) compliance, delayed cases, and key process times. Cases missing the target were analyzed for contributing delays, corrective actions were implemented, and performance was reassessed in subsequent meetings. Sustainability was defined as maintaining Golden Hour compliance at or above the applicable KPI target (≥60% during Cycle 1 and ≥90% from Cycle 2 onward) across consecutive calendar years.

**Figure 1:**
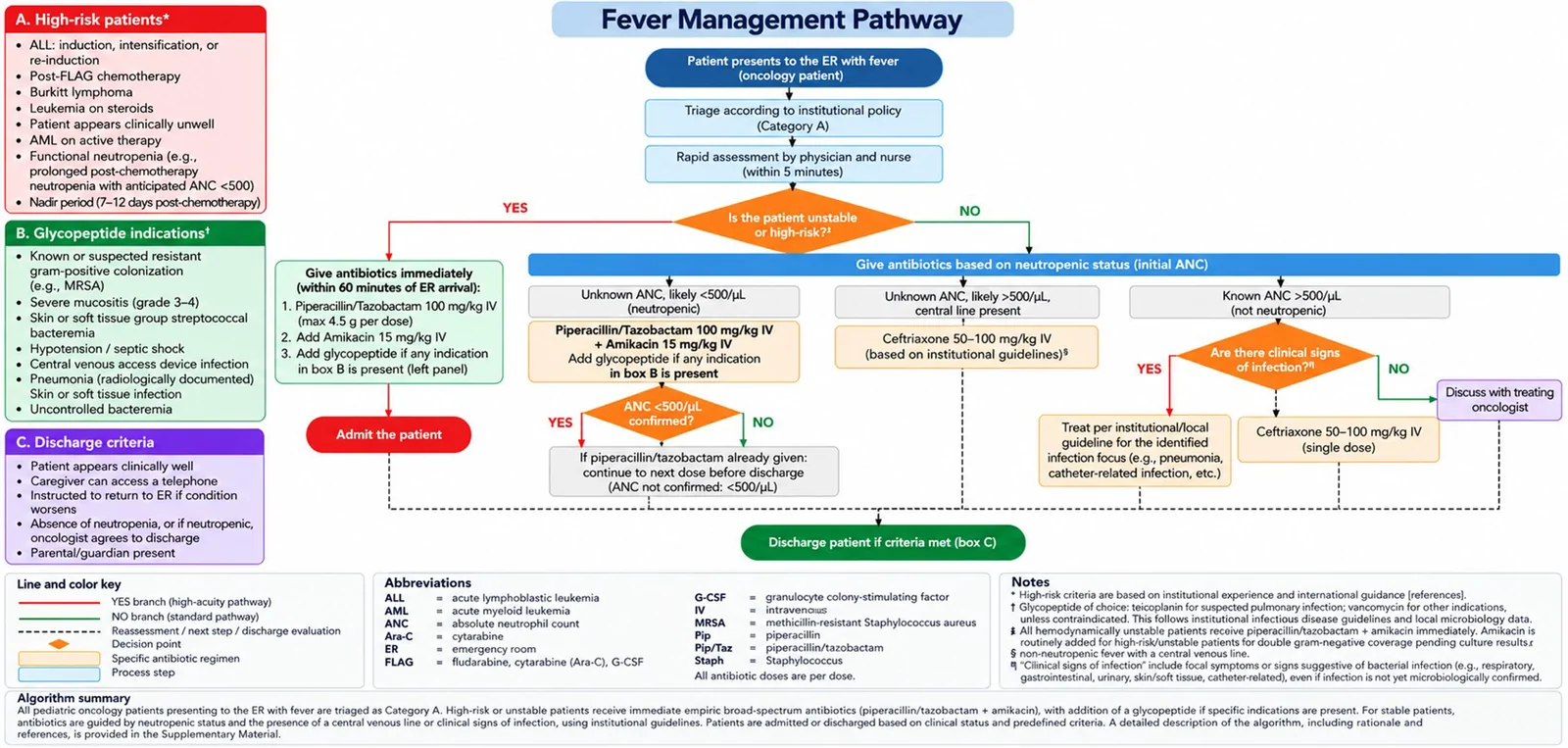
Fever management pathway for children with cancer presenting to the emergency room. The pathway illustrates initial triage and assessment, identification of high-risk or clinically unstable patients, antibiotic selection according to neutropenic status and clinical findings, indications for glycopeptide therapy, and criteria for admission or discharge. ALL, acute lymphoblastic leukemia; AML, acute myeloid leukemia; ANC, absolute neutrophil count; Ara-C, cytarabine; ER, emergency room; FLAG, fludarabine, cytarabine (Ara-C), and granulocyte colony-stimulating factor (G-CSF); G-CSF, granulocyte colony-stimulating factor; IV, intravenous; MRSA, methicillin-resistant Staphylococcus aureus.

### QI framework and team

We applied the Find, Organize, Clarify, Understand, and Select–Plan, Do, Check, and Act (FOCUS–PDCA) framework (Table 1) (19, 20). The QI team included pediatric oncologists, emergency physicians, nurses, pharmacists, and a QI representative. The PDCA cycle comprised four phases: Plan (define the intervention), Do (implement the change), Check (evaluate results), and Act (sustain or adjust improvements). Baseline barriers and corresponding interventions are summarized in Table 2; the aim, key interventions, and compliance outcome for each of the three sequential PDCA cycles are summarized in Table 3.

**Table 1:** Application of the FOCUS–PDCA framework to the pediatric oncology “golden hour” antibiotic project.

FOCUS–PDCA step	Application for the Golden Hour Project
Find	Identified delayed administration of empiric antibiotics for pediatric oncology patients presenting with fever, frequently exceeding the recommended 60-minute (“Golden Hour”) target.
Organize	Established a multidisciplinary team including pediatric oncologists, emergency physicians, emergency nurses, pharmacists, and a quality improvement representative.
Clarify	Mapped the existing process from ER arrival to antibiotic administration to identify workflow steps and delays.
Understand	Key barriers included triage delays, waiting for physician evaluation, pharmacy preparation time, lack of standardized protocols, and variable staff awareness.
Select	Selected a standardized fever management pathway, staff education, defined team roles, and monthly audit and feedback to improve time-to-antibiotic delivery.
Plan–Do–Check–Act	A 6-month staff education and preparation phase was completed before project launch. The pathway was then implemented from July 2019 onward, with monthly review of performance data and delayed cases, followed by iterative actions to sustain improvement

**Table 2:** Baseline barriers and corresponding interventions in the golden hour project.

Baseline barriers	Intervention
Delays in triage prioritization	Automatic Category A triage for febrile oncology patients
Delays in physician assessment	Mandatory physician evaluation within 5 min
Delays in antibiotic ordering	STAT antibiotic orders for high-risk patients
Delays in pharmacy preparation	Pharmacy preparation within 30 min
Delays in nursing administration	Immediate antibiotic infusion upon delivery
Variable staff awareness	Staff education campaign and monthly audit-feedback

**Table 3:** Summary of PDCA implementation cycles.

Cycle	Period	Aim	Key interventions	Compliance outcome
1	July 2019—Aug 2020	Achieve ≥60% Golden Hour compliance and establish baseline performance	Automatic Category-A triage for all febrile oncology patients STAT antibiotic ordering protocol with mandatory physician assessment within 5 min of arrival Pre-ANC antibiotic initiation for high-risk patients Role definitions for each care step (triage nurse → physician → pharmacist → bedside nurse) Monthly audit-feedback meetings initiated	60% target met 2/12 months in 2019 60% target met 8/8 months in 2020 Median PTA fell from 120 min (baseline) toward target range
2	Sept 2020—Dec 2021	Escalate compliance target to ≥90%	Protocol reinforcement for existing staff Targeted retraining of staff hired after initial education campaign Pharmacy preparation workflow refinement to reduce order-to-administration time Corrective actions implemented for any month below target	90% target met 4/4 months (Sep-Dec 2020) and 12/12 months in 2021 Compliance target formally raised from 60% to 90% Median arrival-to-order time: 15 min (down from 53 min at baseline)
3	Jan 2022—Dec 2024	Sustain ≥90% compliance over the long term	Continuous onboarding education for new staff Integration of Golden Hour pathway into institutional clinical guidelines Monthly KPI tracking maintained throughout Immediate review and targeted response triggered by any non-compliant month	90% met 9/12 months in 2022, 11/12 in 2023, 8/12 in 2024 Non-compliant months remained ≥85% throughout Median PTA: 50 min overall; 45 min in 2022 (lowest recorded)
4	2025 onwards (planned)	Reduce order-to-administration time from 32 min to ≤15 min; achieve total PTA ≤30 min in ≥80% of visits	Ward-level pre-stocking of cefepime and piperacillin-tazobactam in the oncology ER medication cabinet Pharmacist-initiated preparation triggered automatically on STAT order entry, without waiting for nursing confirmation Dedicated oncology ER antibiotic preparation checklist, reviewed at monthly KPI meetings	*Planned — not yet implemented* **Target:** total PTA ≤30 min in ≥80% of FN visits **Rationale:** *≤30 min associated with lowest 28-day mortality in adult FN (Rosa* et al.*, ref 30)*

PDCA, Plan–Do–Check–Act; PTA, presentation-to-antibiotic time; ANC, absolute neutrophil count; KPI, Key Performance Indicator; STAT, immediately; min, minutes.

### Aims

The global aim was to improve the timeliness of antibiotic administration for children with FN presenting to the ER. The SMART aim was to increase Golden Hour compliance from a pre-intervention baseline of 22% to ≥60% within the first two years, with a pre-specified plan to escalate the target to ≥90% once sustained achievement was confirmed.

### Fever management pathway

The team developed a fever management pathway detailing the process from ER arrival to either admission (if neutropenic) or discharge (if low risk) (Figure 1). High-risk patients — those in whom a delay in antibiotic initiation was expected to result in serious complications — received antibiotics immediately without waiting for absolute neutrophil count (ANC) confirmation (Figure 1).

The pathway standardized four sequential steps: 1) Triage: children with cancer presenting with fever are automatically classified as Category-A (highest priority) within the existing five-tier triage system, triggering immediate evaluation. 2) Physician assessment: physicians were required to assess the patient within 5 min of arrival; if high-risk criteria were met, antibiotics were ordered immediately and marked STAT. 3) Pharmacy preparation: the pharmacy prepared STAT antibiotics within 30 min and notified ER nursing staff upon completion. 4) Nursing administration: nurses initiated the infusion immediately upon receiving the prepared medication.

### Education

Six months before project launch, a structured educational campaign was delivered to all ER-based providers involved in FN management: emergency physicians, pediatric oncology nurses, bedside nurses, and clinical pharmacists. A QI representative and a senior pediatric oncologist led training through didactic lectures, case-based discussions, and bedside demonstrations. Training covered FN recognition, Category-A triage assignment, STAT ordering (including pre-ANC initiation for high-risk patients), and defined team roles at each care step. Data collection personnel underwent separate targeted training assessed by direct observation.

### Ongoing monitoring

Monthly audit-and-feedback meetings provided performance monitoring and reinforcement throughout all PDCA cycles, focusing on triage compliance, laboratory turnaround, and pharmacy preparation times.

### Measures

The primary measure was total PTA time — the interval from ER arrival to initiation of the first antibiotic dose. When a patient received multiple antibiotics, the time to the first administered dose was used (Figure 2A). Two process measures were tracked: arrival-to-order time (ER registration to physician antibiotic order entry) and order-to-administration time (order entry to start of first infusion, reflecting pharmacy preparation and nursing administration). Balancing measures monitored for medication errors and workflow disruptions.

**Figure 2:**
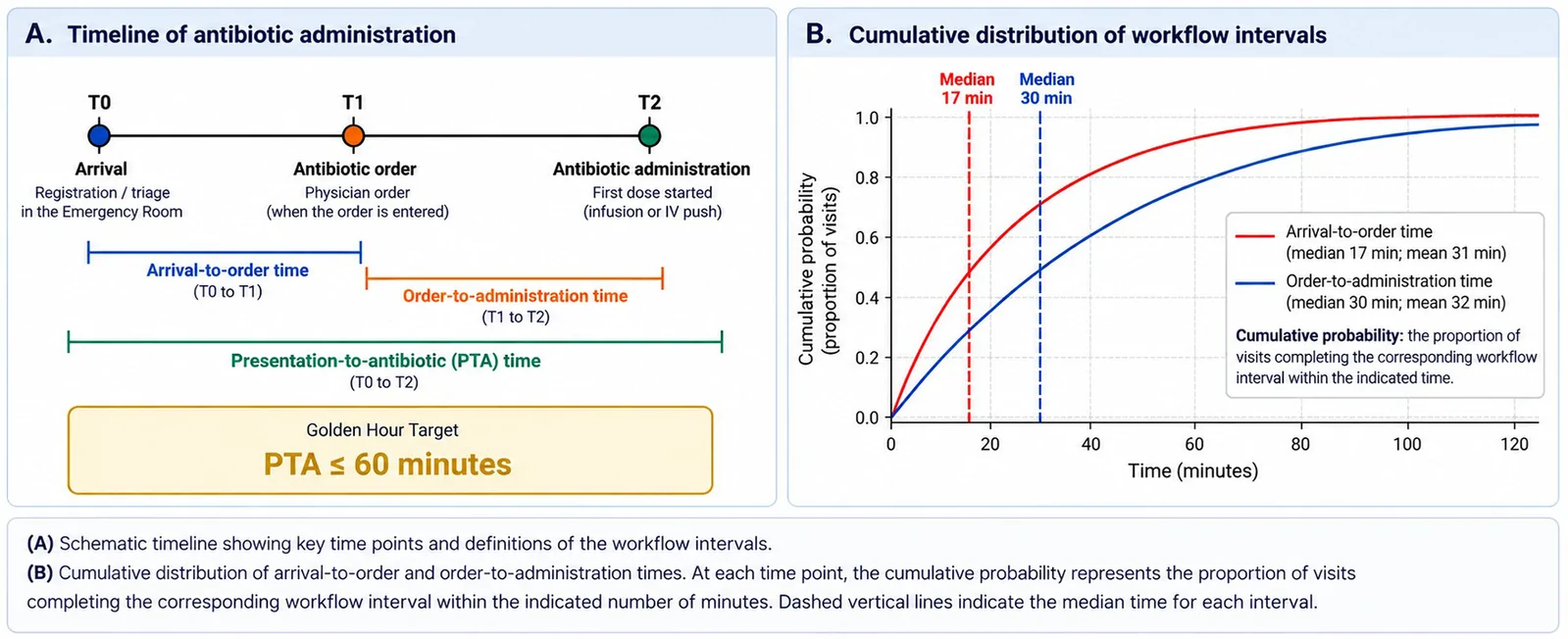
**(A)** Timeline of antibiotic administration illustrating the components of presentation-to-antibiotic (PTA) time and the 60-minute “golden hour” target. **(B)** Cumulative distribution of arrival-to-order and order-to-administration times. Cumulative probability represents the proportion of visits completing each workflow interval within the indicated time. Dashed vertical lines indicate the median time for each interval.

### Data collection

An electronic data collection sheet captured the date of visit, arrival time, antibiotic order time, and antibiotic administration time, along with patient demographics (age, sex) and clinical characteristics (cancer type, ANC). High-risk status was assessed clinically at presentation according to the predefined institutional criteria but was not captured as a separate structured variable in the QI dataset. An assigned nurse completed the sheet immediately after each ER encounter. Data were stored on a password-protected secure system. A quality office coordinator generated summary figures and graphs from the collected data.

### Data analysis

As this was a continuous, real-time QI initiative that captured all consecutive eligible ER visits during the study period, the analysis represented a full census rather than a sampled cohort; therefore, no formal *a priori* sample size or power calculation was performed. Statistical significance was defined as a two-sided *p* < 0.05, with 95% confidence intervals reported for effect estimates where applicable. Statistical analyses were performed by I.S., a co-author with formal training and experience in biostatistical analysis, and were reviewed by the study team. Continuous variables are reported as means with standard deviations or medians with interquartile ranges, as appropriate; categorical variables as frequencies and percentages. Descriptive statistics summarized patient demographics, clinical characteristics, and the three key time intervals (arrival-to-order, order-to-administration, and total PTA time).

Monthly and annual Golden Hour compliance trends were examined, including KPI achievement rates by year. Arrival times were categorized as working hours, after-hours, or weekends, and compliance rates compared across these groups. A pre-specified subgroup analysis compared compliance before and after September 2020, when the KPI target was raised from 60% to 90%.

Patient visits were stratified by Golden Hour compliance (PTA ≤60 vs. >60 min). Because individual patients could contribute more than one FN episode during the study period, the potential within-patient correlation of repeated encounters was accounted for using generalized estimating equations (GEE), with patient ID specified as the clustering variable. Between-group differences were assessed using chi-square tests for categorical variables and independent-samples t-tests or Mann–Whitney U tests for continuous variables, depending on distributional assumptions. Multivariable logistic regression identified patient- and visit-level factors independently associated with non-compliance; covariates included patient age, ANC at presentation, cancer type, and time of ER arrival (working hours vs. after-hours or weekends).

Thirty-day all-cause mortality was evaluated as an exploratory secondary clinical outcome. Mortality data were retrieved from hospital medical records and the institutional cancer registry. Logistic regression assessed the association between Golden Hour compliance and 30-day mortality, reported as odds ratios (ORs) with 95% confidence intervals. A pre-specified exploratory subgroup analysis was performed in leukemia patients, given their higher baseline risk of infection-related complications.

## Results

### Pre-intervention cohort and baseline performance

During the pre-intervention period (December 2018 to June 2019), 119 patients accounted for 158 FN episodes. The median age was 12 years (IQR, 10–15), 69 patients (58.0%) were male, and the median number of FN visits per patient was 1 (range, 1–4). Median PTA was 120 min (IQR, 97–143), Golden Hour compliance (PTA ≤60 min) was 22%, arrival-to-order time was 53 min, and order-to-administration time was 32 min. Baseline demographic and clinical characteristics of the pre-intervention and intervention cohorts, including underlying diagnoses, are compared in Table 4. The intervention-period results are summarized below according to cohort characteristics, treatment-timing measures, compliance, and clinical outcomes.

**Table 4:** Baseline demographic and clinical characteristics: intervention-period cohort versus pre-intervention control cohort.

Characteristic	Intervention-period cohort (July 2019–December 2024)	Pre-intervention control cohort (December 2018–June 2019)
Patients, N	697	119
ER visits for FN, N	1,164	158
Visits per patient, median (range)	1 (1–11)	1 (1–4)
Age, years, median (IQR)	4.7 (2.6–9.2)	12 (10–15)
Male sex, *n* (%)	377 (54%)	69 (58.0%)
ANC at presentation, cells/μL, median (IQR)	50 (0–180)	89 (16.2–249.3)
Diagnosis, *n* (%)		
B-lymphoblastic leukemia/lymphoma	291 (41.8%)	44 (37.0%)
T-cell acute lymphoblastic leukemia	64 (9.2%)	14 (11.8%)
Neuroblastoma	57 (8.2%)	12 (10.1%)
Nephroblastoma	49 (7.0%)	4 (3.4%)
Other diagnoses	236 (33.9%)	45 (37.8%)

Statistical comparison: The pre-intervention cohort was significantly older than the intervention-period cohort (median age, 12 vs. 4.7 years; *p* < 0.001), whereas sex distribution (*p* = 0.430) and the overall distribution of underlying diagnoses (*p* = 0.370) did not differ significantly between cohorts. FN, febrile neutropenia; ER, emergency room; ANC, absolute neutrophil count; IQR, interquartile range. Age, sex, and diagnosis are reported at the patient level; ANC is reported at the episode level. Other diagnoses include all diagnoses not listed separately.

### Intervention cohort characteristics

During the intervention period (July 2019 to December 2024), 697 children with cancer and FN were included, accounting for 1,164 FN events. Median visits per patient were one (range, 1–11). Median age was 4.7 years (IQR, 2.6–9.2 years), 377 patients (54%) were male, and median ANC at presentation was 50 cells/μL (IQR, 0–180). Hematologic malignancies predominated: B-lymphoblastic leukemia/lymphoma (291 patients, 41.8%), and T-cell acute lymphoblastic leukemia (64, 9.2%) were the most frequent diagnoses. Among solid tumors, neuroblastoma (57, 8.2%) and nephroblastoma (49, 7.0%) were most common. Patients with AML were underrepresented in this cohort, because they receive intensive chemotherapy and are at high risk for profound neutropenia and severe infection. Our current institutional practice is to monitor complete blood count (CBC)ICU.

every other day during expected neutropenia and electively admit patients once ANC falls below 300 cells/µL. This approach was adopted after prior experience with late ER presentations complicated by severe infection or septic shock, including fatal cases.

### Antibiotic timeliness and golden hour compliance

Median PTA time improved from 120 min (IQR, 97–143) at baseline to 50 min (IQR, 40–65) across the full intervention period, reaching 45 min (IQR, 36–58) by 2022 (*p* < 0.01) (Figure 3). The improvement was driven by a reduction in arrival-to-order time, which fell from 53 min at baseline to 15 min post-intervention (*p* < 0.01). Order-to-administration time remained stable throughout, with a median of 32 min (IQR, 25–40) (Figure 2B), indicating that pre-intervention delays originated in early clinical evaluation and antibiotic ordering rather than in pharmacy preparation or nursing administration.

**Figure 3:**
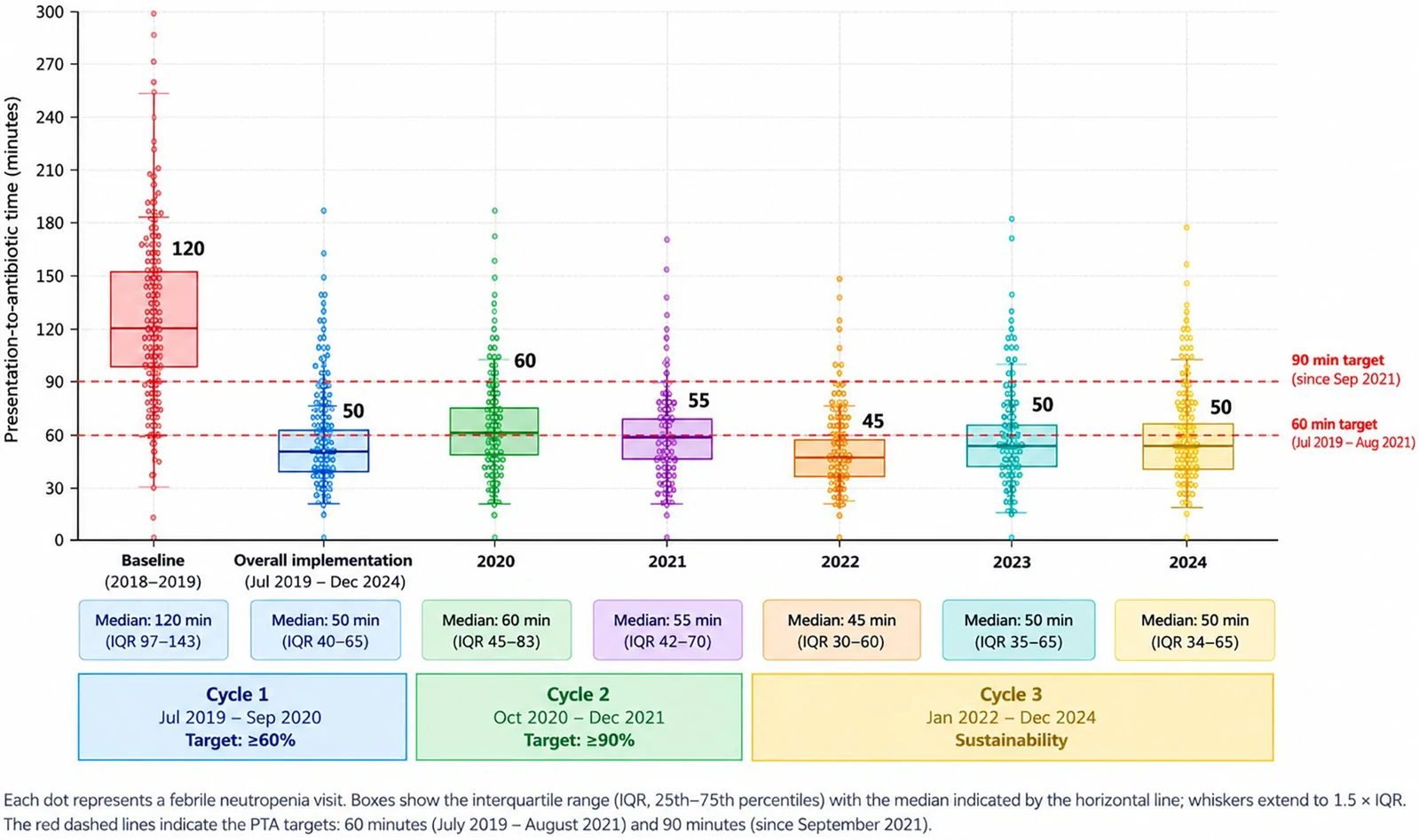
Presentation-to-antibiotic time during the study period. Each dot represents a febrile neutropenia visit. Boxes represent the interquartile range (IQR, 25th–75th percentiles), with the median indicated by the horizontal line; whiskers extend to 1.5 × IQR. The red dashed horizontal lines indicate presentation-to-antibiotic time thresholds of 60 and 90 min. Boxes below the graph indicate the three quality-improvement implementation cycles.

The initial 60% compliance target was met in 2 of 12 months in 2019 and in all 8 months from January through August 2020. The target was subsequently increased to 90% in September 2020. Following this escalation, the 90% compliance target was achieved in all 4 remaining months of 2020, all 12 months in 2021, 9 of 12 months in 2022, 11 of 12 months in 2023, and 8 of 12 months in 2024. In months when the target was not met, compliance remained ≥85%.

### Factors associated with non-compliance

Of 1,164 visits, 138 (11.9%) did not meet the 60-minute target; no visit exceeded 120 min. Delayed visits had significantly longer arrival-to-order times than compliant visits (median 21.9 vs. 4.8 min; *p* < 0.001). Non-compliance was not associated with diagnosis, ANC at presentation, age, or time of arrival (working hours vs. after-hours vs. weekends; *p* > 0.05 for all).

### Thirty-day mortality

Thirty-day all-cause mortality within 30 days of the index ER visit varied by diagnosis. Among patients with leukemia, five deaths occurred (5/460, 1.1%); among patients with brain tumors, one death occurred (1/30, 3.3%; medulloblastoma); among those with bone tumors, one death occurred (1/54, 1.9%; Ewing sarcoma); and among other solid tumors, one death occurred (1/135, 0.7%; neuroblastoma); no deaths occurred among patients with lymphoma or retinoblastoma (Figure 4). In an exploratory analysis, Golden Hour compliance was associated with lower 30-day mortality (OR 0.18, 95% CI 0.03–0.78; *p* = 0.036). In a pre-specified exploratory subgroup analysis restricted to patients with leukemia, the point estimate was attenuated, and the association did not reach statistical significance (OR 0.38, 95% CI 0.05–2.32; *p* = 0.291).

**Figure 4:**
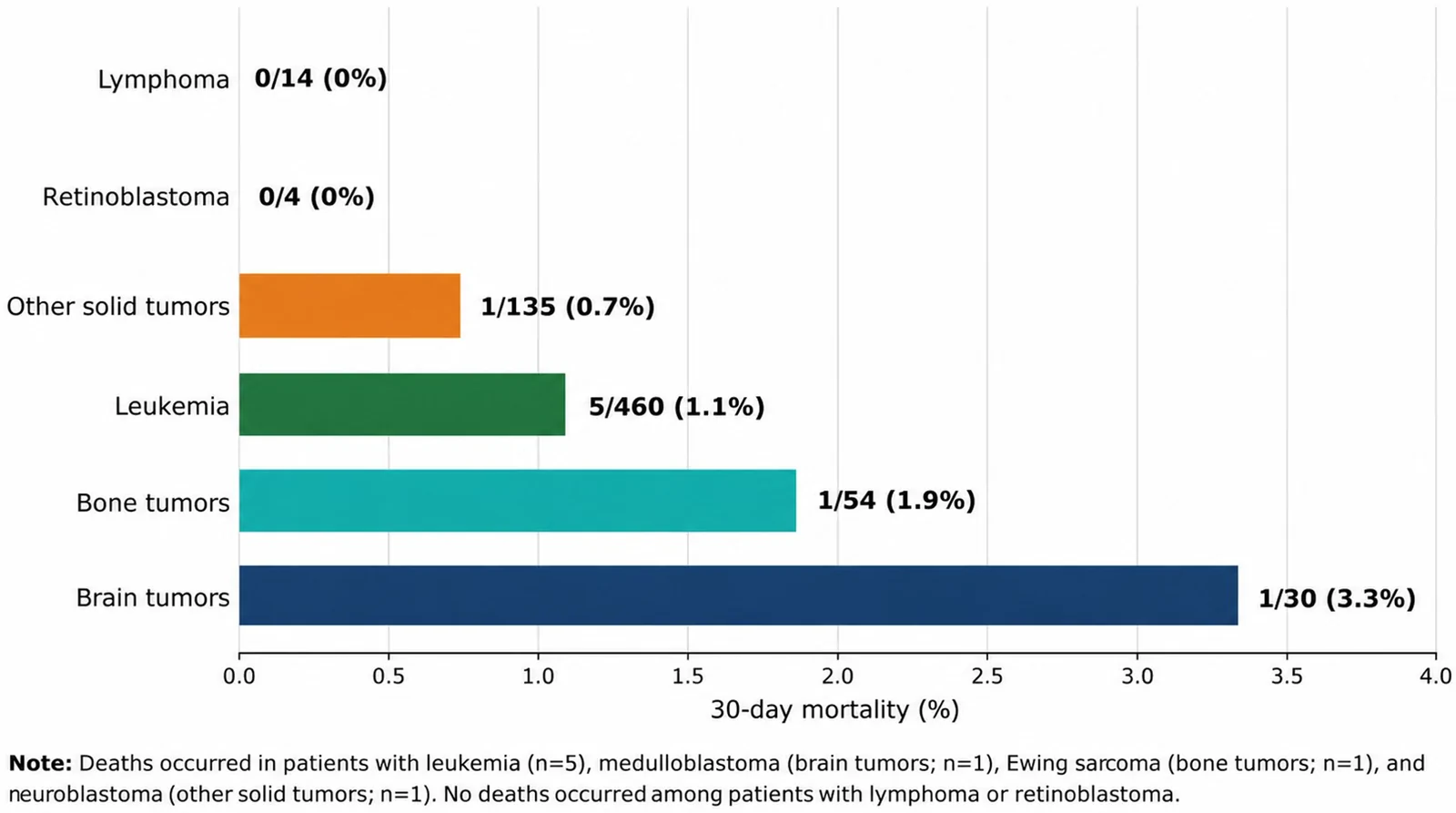
Thirty-day all-cause mortality by malignancy type among children presenting with febrile neutropenia. Values are presented as deaths/total visits (%). Deaths occurred among patients with leukemia (*n* = 5), medulloblastoma (brain tumors; *n* = 1), Ewing sarcoma (bone tumors; *n* = 1), and neuroblastoma (other solid tumors; *n* = 1). No deaths occurred among patients with lymphoma or retinoblastoma.

## Discussion

At KHCC, implementation of the “Golden Hour” QI project led to a rapid and sustained increase in Golden Hour compliance, from 22% at baseline to 85%–90% across 5.5 years and 1,164 FN visits — exceeding 90% adherence in multiple calendar years. Unlike many QI initiatives that achieve early gains but lose momentum over time, this program maintained high performance over the full observation period. This long-term success highlights the importance of key strategies such as standardized processes, comprehensive staff training, readily accessible resources, and continuous audit and feedback. Together, these elements fostered a lasting change in clinical practice and embedded a culture of timely, high-quality care within our pediatric emergency oncology services (10, 21, 22). The interventions employed at KHCC — automatic Category-A triage, pre-ANC antibiotic initiation for high-risk patients, STAT ordering protocols, and monthly audit-and-feedback cycles — mirror the strategies identified as effective in high-income country (HIC) settings, yet were implemented here without additional staffing, technology, or budget.

The pre-intervention cohort was smaller and covered a shorter period, and patients were older than those in the intervention cohort (median age, 12 vs. 4.7 years). However, the distribution of underlying diagnoses was similar between cohorts (*p* = 0.370; Table 4). Despite this age difference, the marked reduction in PTA and sustained increase in Golden Hour compliance support improvement in care processes following implementation of the QI intervention. At baseline, KHCC's median PTA of 120 min (IQR 97–143) and 22% compliance were comparable to pre-intervention figures reported in HIC QI studies: 97 min (35% compliance) at Children's of Alabama, 99 min at the Volpe et al. cohort, and 83 min at the Yoshida et al. pediatric ED (11, 12, 23–26). Following the intervention, KHCC's median PTA of 50 min and sustained compliance of 85%–90% were on par with — and in several cases exceed — post-intervention results from those HIC centers, achieved in a low- and middle-income country (LMIC) setting. This places KHCC among the largest and longest-running single-center FN QI cohorts in the LMIC literature. To our knowledge, this is the first prospective Golden Hour QI program reported from the Middle East and North Africa (MENA) region, where pediatric oncology FN data remain limited and LMIC-adapted protocols are critically needed.

These results are consistent with prior evidence supporting prompt antibiotic administration in FN (25, 27, 28). Salstrom et al. achieved near-complete compliance at a U.S. pediatric oncology center after implementing Lean/Six Sigma processes (29). Cash et al. reduced the median time to antibiotic (TTA) administration from 154 to 95 min following implementation of a standardized ER process for children with FN (24). A QI initiative at Hospital General Tijuana increased timely antibiotic administration from 50% to 88% using locally adapted interventions (10). Together, these studies and the present data suggest that the core principles of early FN recognition and rapid antibiotic response are applicable across resource settings. The decline in compliance to 85.8% in 2024—the first decline after several years of sustained improvement—warrants closer monitoring. Potential contributors include staff turnover and competing operational demands during periods of high patient volume, although the specific causes could not be determined from the available data. To support sustainability, the QI team reinforced case-level review of non-compliant visits during monthly audit-feedback meetings, targeted retraining of newly onboarded staff, and incorporation of Golden Hour practices into departmental onboarding. Planned Cycle 4 interventions targeting the order-to-administration interval are intended to further strengthen and sustain performance.

The sub-process data reveal where the problem actually lived — and where it did not. Arrival-to-order time collapsed from 53 min at baseline to 15 min after intervention (*p* < 0.01), driven by triage reclassification and mandatory physician assessment within 5 min of arrival. Order-to-administration time, by contrast, remained essentially unchanged throughout the 5.5-year project period (median 32 min, IQR 25–40) — meaning that pharmacy preparation and nursing infusion were never the rate-limiting step at KHCC. This distinction carries a practical implication for other centers considering a similar initiative: in settings where the pre-ordering process is already well managed, the same triage and physician-assessment interventions used here will likely yield diminishing returns, and the focus should instead shift to pharmacy workflow — stocking antibiotics on the ward, implementing nurse-initiated preparation, or enabling bedside dispensing. Volpe et al. and similar multidisciplinary QI programs have confirmed that the pre-ordering phase is the most common bottleneck across centers (26), Koenig et al. similarly emphasized the importance of analyzing the individual components of time to antibiotics rather than relying only on the total interval (30), suggesting the KHCC finding is generalizable. The stable order-to-administration time at KHCC is therefore less a success than an unfinished agenda: reducing it further, toward a target of 15 min, would create the headroom needed to achieve a 30 min total PTA — the interval associated with the greatest mortality benefit in adults (8).

Notably, among the 1,164 FN visits captured over 5.5 years, no single visit exceeded 120 min from arrival to antibiotic administration, minimizing exposure to the prolonged treatment delays associated with higher mortality risk in prior FN cohorts. In exploratory analysis, Golden Hour compliance was associated with lower 30-day mortality (OR 0.18, 95% CI 0.03–0.78; *p* = 0.036). However, given the small number of mortality events, this finding should be interpreted cautiously and does not establish a causal relationship between timely antibiotic administration and mortality. Several studies have reported an association between shorter time to first antibiotic administration in children with FN and reduced mortality and morbidity (8, 31, 32). Fletcher et al. revealed that TTA interval of ≤60 min, when compared to a TTA of 61–120 min, was associated with a lower rate of the composite adverse outcome (31). Rosa et al. showed that early antibiotic administration was associated with higher survival rates in the context of FN with 28-day mortality rates of 3% if antibiotic given in ≤30-minutes versus 18.1% if given 31–60 min (*p* < 0.0002) (8). However, recent pediatric evidence is less definitive. Salomon et al., in an international individual-patient-data meta-analysis, found no clear association between shorter time to antibiotics and improved clinical outcomes, suggesting that the relationship may vary according to patient risk and clinical context (33). Multiple studies have demonstrated that administering the first dose of antibiotics within 30 min of patient presentation is feasible and achievable (34, 35). Our study primarily emphasized administering antibiotics within 60 min from presentation to the ER. Based on the results highlighted in the previously mentioned studies, a follow-on QI cycle aimed at reducing the TTA target to 30 min would be a logical next step.

The order-to-administration interval (median 32 min) did not improve across 5.5 years and three PDCA cycles, indicating a structural rather than behavioral bottleneck. A Cycle 4 targeting this segment should test three low-cost interventions: (1) ward-level pre-stocking of cefepime and piperacillin-tazobactam to eliminate dispensing transit time; (2) pharmacist-initiated preparation triggered automatically by the STAT order, without waiting for nursing confirmation; and (3) a pharmacy preparation checklist reviewed at monthly KPI meetings. These measures could reduce order-to-administration time to ≤15 min, enabling a total PTA of ≤30 min — the interval associated with the greatest mortality benefit in adult FN (8).

This study has several strengths. As one of the largest and longest-running single-center FN QI cohorts reported from an LMIC, it demonstrates that structured interventions can achieve and sustain compliance with international benchmarks over 5.5 years in a resource-constrained setting. The shift from 22% to 85%–90% compliance across 1,164 visits, without additional staff, technology, or funding, provides a replicable proof of concept for other LMIC centers. The prospective design, continuous data collection, and integration of sub-process interval analysis further strengthen the internal validity of the findings.

This study has several limitations. First, the single-center before-and-after design without a concurrent control group limits causal inference, as temporal changes or other unmeasured factors may have contributed to the observed improvements. The single-center setting may also limit generalizability to institutions with different resources or patient populations. Second, although the dataset spans 5.5 years, sustainability beyond this period remains uncertain; planned Cycle 4 interventions targeting the order-to-administration interval and continued incorporation of Golden Hour competency into onboarding for new staff (Table 3) are intended to help preserve these gains. Third, causality between process improvement and patient-centered outcomes, such as reduced intensive care unit (ICU) admissions or improved survival, cannot be established, as uncontrolled factors including concurrent changes in staffing, patient volume, or institutional culture may have contributed to the observed improvements. Fourth, documentation errors in electronic records may have introduced bias into timestamp-derived calculations. In addition, high-risk status was not captured as a structured variable, precluding reliable estimation of the proportion of high-risk episodes and comparison of mortality or ICU admission between high- and non-high-risk groups. Fifth, no medication errors attributable to the accelerated antibiotic process were identified across 1,164 visits; however, the study was not powered to detect rare safety events. Sixth, ER overcrowding was not systematically measured as a balancing measure, representing a gap that future projects should address using standardized crowding metrics. Seventh, the exploratory leukemia subgroup mortality analysis should be interpreted with caution. Golden Hour compliance was not associated with a statistically significant reduction in 30-day mortality within this subgroup (OR 0.38, 95% CI: 0.05–2.32, *p* = 0.291), likely reflecting limited statistical power; the subgroup contains few mortality events, combines AML and acute lymphoblastic leukemia (ALL) patients despite their markedly different infection-risk profiles, and is subject to potential selection bias because AML patients are predominantly managed as inpatients at KHCC and therefore rarely present to the ER, resulting in their underrepresentation in this cohort and limiting the generalizability of the findings to AML specifically. Haeusler et al. similarly found no definite evidence that a PTA of ≥60 min — but within two hours — significantly increased adverse outcomes in pediatric FN, suggesting the relationship between antibiotic timing and mortality may vary by disease subtype and baseline risk (5). A leukemia-specific analysis, stratified by AML versus ALL, in a larger multicenter cohort is needed. Eighth, the empiric antibiotic regimen was not guided by a systematic, continuously updated local antimicrobial-resistance surveillance program; formal integration of institutional antibiogram data into the fever pathway is planned as a future refinement.

In conclusion, implementation of this structured QI initiative using the FOCUS–PDCA framework was associated with substantial and sustained improvement in antibiotic timeliness for pediatric FN at a single LMIC center. Golden Hour compliance increased from 22% to 85%–90%, and median PTA time decreased from 120 to 50 min over 5.5 years following three low-cost, behavior-based interventions: automatic Category-A triage for all children with cancer presenting with fever, pre-ANC antibiotic initiation for high-risk cases, and monthly KPI audit-feedback cycles. The initiative was implemented within existing workflows at no additional cost and is now embedded in KHCC's institutional guidelines as standard of care. As the first such initiative reported from a Middle Eastern LMIC center, these findings may provide a model for other pediatric oncology programs across the MENA region operating within similar resource constraints. However, the single-center before-and-after design without a concurrent control group limits causal inference, and the mortality findings should be considered exploratory given the small number of events.

## Data Availability

The raw data supporting the conclusions of this article will be made available by the authors, without undue reservation.
